# Evaluating variants classified as pathogenic in ClinVar in the DDD Study

**DOI:** 10.1038/s41436-020-01021-9

**Published:** 2020-11-05

**Authors:** Caroline F. Wright, Ruth Y. Eberhardt, Panayiotis Constantinou, Matthew E. Hurles, David R. FitzPatrick, Helen V. Firth, on behalf of the DDD Study

**Affiliations:** 1 Institute of Biomedical and Clinical Science, University of Exeter Medical School, Exeter, UK; 2 Wellcome Sanger Institute, Wellcome Genome Campus, Hinxton, Cambridge, UK; 3 Queen Elizabeth University Hospital, Glasgow, UK; 4 MRC Human Genetics Unit, MRC Institute of Genetics and Molecular Medicine, University of Edinburgh, Western General Hospital, Edinburgh, UK; 5 East Anglian Medical Genetics Service, Cambridge University Hospitals NHS Foundation Trust, Cambridge Biomedical Campus, Cambridge, UK

**Keywords:** variant interpretation, exome sequencing, reanalysis, genomic medicine, developmental disorders

## Abstract

**Purpose:**

Automated variant filtering is an essential part of diagnostic genome-wide sequencing but may generate false negative results. We sought to investigate whether some previously identified pathogenic variants may be being routinely excluded by standard variant filtering pipelines.

**Methods:**

We evaluated variants that were previously classified as pathogenic or likely pathogenic in ClinVar in known developmental disorder genes using exome sequence data from the Deciphering Developmental Disorders (DDD) study.

**Results:**

Of these ClinVar pathogenic variants, 3.6% were identified among 13,462 DDD probands, and 1134/1352 (83.9%) had already been independently communicated to clinicians using DDD variant filtering pipelines as plausibly pathogenic. The remaining 218 variants failed consequence, inheritance, or other automated variant filters. Following clinical review of these additional variants, we were able to identify 112 variants in 107 (0.8%) DDD probands as potential diagnoses.

**Conclusion:**

Lower minor allele frequency (<0.0005%) and higher gold star review status in ClinVar (>1 star) are good predictors of a previously identified variant being plausibly diagnostic for developmental disorders. However, around half of previously identified pathogenic variants excluded by automated variant filtering did not appear to be disease-causing, underlining the continued need for clinical evaluation of candidate variants as part of the diagnostic process.

## INTRODUCTION

Over the past decade, the diagnosis of rare pediatric diseases has been transformed by the application of next-generation sequencing.^1^ In particular, the widespread use of exome sequencing in family trios (proband, mother, and father) has catalyzed disease gene discovery^2^ and improved diagnostic yields.^3^ Due to the enormous amount of variation present in every genome,^4^ bioinformatics pipelines have developed alongside next-generation sequencing to facilitate genomic analysis.^5^ These automated workflows aim to exclude the vast majority of benign variants in the genome while prioritizing those that are plausibly pathogenic. However, like all tests, there is a tradeoff between sensitivity and specificity. Hard thresholds are usually applied to exclude all but a small minority of variants, without which diagnostic services would be overwhelmed by false positive results, especially for highly genetically heterogeneous and incompletely penetrant disorders. Unfortunately, these hard cutoffs can sometimes inadvertently exclude important diagnoses, leading to false negative results.

Cognizant of this problem, we were motivated to investigate whether some previously identified pathogenic variants may be being routinely excluded by standard clinical variant filtering pipelines. Although numerous studies have shown the value of reanalysing exome sequence data,^6,7^ the focus of these studies has primarily been novel disease genes that were discovered after the original analysis, rather than incorrect variant filtering. Using exome sequence data from the Deciphering Developmental Disorders (DDD) study,^8^ here we show that around 16% of potentially relevant previously reported pathogenic variants are excluded by the standard DDD clinical variant filtering pipeline.^9^ Clinical evaluation of these excluded variants suggests that around half may be miscategorized in ClinVar, while the other half provided a full or partial diagnosis for 107 (0.8%) probands in the DDD study.

## MATERIALS AND METHODS

The DDD study (www.ddduk.org) recruited probands with severe previously undiagnosed developmental disorders, and their parents, from 24 National Health Service (NHS) regional genetics services around the UK and Ireland. Clinical information and quantitative growth data were collected systematically via the DECIPHER database,^10^ and probands were phenotyped by their referring consultant clinical geneticist using the Human Phenotype Ontology (HPO).^11^ Exome sequencing and microarray analysis was performed from saliva and blood-extracted DNA and variants called and annotated as described previously.^8^ Variants were evaluated for clinical feedback using a curated developmental disorder gene-to-phenotype database (DDG2P)^12^ and a bespoke series of variant filtering rules described previously^7,9^ (see https://github.com/jeremymcrae/clinical-filter); in brief, variants are excluded based on minor allele frequency (MAF), predicted consequence, and genotype or inheritance inconsistent with either the family history or the allelic requirement of the DDG2P gene–disease pair.

We obtained a list of clinically annotated variants from ClinVar^13^ (*clinvar_variant_summary.txt.gz* and *clinvar_20190923.vcf.gz*, downloaded from https://www.ncbi.nlm.nih.gov/clinvar/ on 24 September 2019). We restricted the data set to germline variants in GRCh37 that were annotated with the status of pathogenic or likely pathogenic (P/LP) without any conflicting interpretations and had a review status of one or more gold stars (*n* = 59,240 variants). We further restricted the variants to those in genes annotated as having robust disease association in a clinically curated panel of developmental disorder genes, DDG2P (downloaded from https://www.ebi.ac.uk/gene2phenotype/ on 18 April 2019), and searched in the exome sequence data from 13,462 DDD probands (including 9859 parent–offspring trios and 3603 singleton probands) for variants with appropriate zygosity, i.e., heterozygous in monoallelic genes, homozygous or compound heterozygous in biallelic genes, and hemizygous in X-linked dominant genes. We then eliminated the following classes of problematic variants: internal MAF > 0.0005 in monoallelic and X-linked genes and MAF > 0.005 in biallelic genes, genotype quality (GQ) < 30, recurrent indels present in >8 unrelated probands, and double heterozygotes in biallelic genes in singleton probands (where we were unable to determine the phase of the variants).

**Fig. 1:**
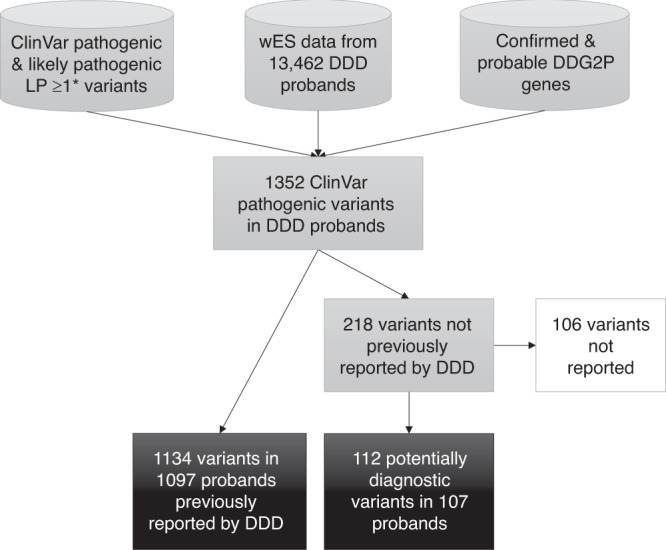
Flowchart of variant selection method and results. *DDD* Deciphering Developmental Disorders study, *DDG2P* developmental disorder gene-to-phenotype database, *ES* exome sequencing, *LP* likely pathogenic.

We parsed the ClinVar P/LP DDD variants into two sets: (1) variants previously reported by the DDD study, and (2) variants not previously reported by the DDD study (see Fig. 1). Variants with low read depth or annotated as de novo were visually assessed using IGV to determine validity and evaluate potential mosaicism in the child or either parent, then likely false positives were excluded. The remaining variants were reviewed for phenotype fit by the DDD clinical review panel, including two consultant clinical geneticists, and variants that might explain all or part of a proband’s phenotype were reported to the referring clinician via DECIPHER for clinical evaluation, validation, and discussion with the family if appropriate. Reported variants linked to individual phenotypes are publicly available via DECIPHER.

### Ethics statement

All DDD data was analyzed and shared in accordance the UK Research Ethics Committee approval (10/H0305/83, granted by the Cambridge South REC, and GEN/284/12 granted by the Republic of Ireland REC). Informed consent was obtained from all subjects.

## RESULTS

We found 37,794 P/LP variants with a ≥1-star rating in ClinVar in diagnostic DDG2P genes. Of these, we detected 1352 (3.6%) variants in 13,462 DDD probands (Fig. 1), of which 1134 (83.9%) variants in 1097 probands had previously been reported to DDD clinical teams as likely diagnoses.^7,9,14,15^ The remaining 218 (16.1%) variants in 213 probands had not previously been reported and were excluded from the standard variant filtering pipeline for a variety of reasons relating to variant quality, predicted consequence, allele frequency, and inheritance (Table 1). Interestingly, 97 variants were excluded in trios due to being inherited from an apparently unaffected parent.

**Table 1:** Reasons ClinVar pathogenic and likely pathogenic variants were excluded from the DDD automated clinical variant filter.

Variant type	Reasons for exclusion	*N* variants (% reported)
De novo variants	Low read depth and/or allele balance	16 (88%)
Heterozygous variants in monoallelic genes	Inherited from apparently unaffected parent	97 (25%)
	ExAC allele count >5	15 (47%)
	MAF > 0.0001 and inheritance unknown	11 (18%)
X-chromosome variants	Heterozygotes in males (due to mosaicism or XXY aneuploidy)	3 (67%)
	Heterozygotes in females in hemizygous genes without confirmed de novo status	3 (67%)
	Inherited in X-linked overdominance gene	2 (100%)
Homozygous variants in biallelic genes	Uniparental inheritance (caused by uniparental disomy)	2 (100%)
Missense variants inherited from unaffected parent or with unknown inheritance	PolyPhen-2^20^ predicted variant to be benign	41 (88%)
Noncoding variants	Consequence predictions (splice region, 5’UTR, 3’UTR, intron, synonymous)	22 (64%)
Variants in genes with imprinting mechanism	Not inherited (de novo or inheritance unknown)	3 (100%)
Variants in gene with mosaic mechanism	Not de novo (inheritance unknown)	1 (100%)
Compound heterozygous variants in biallelic genes	Other variant failed one reason above	2 (100%)
**Total**	**-**	**218 (51%)**

The number of variants refers to the total number of variants detected in 13,462 DDD probands, and the percentage (in parentheses) refers to the proportion of each class that we reported as plausibly diagnostic following clinical review.

*DDD* Deciphering Developmental Disorders study, *MAF* minor allele frequency, *UTR* untranslated region.

These 218 variants were reviewed by two experienced consultant clinical geneticists for plausible fit between the recorded phenotype in the proband and the expected phenotype for the disorder. Following this clinical review process, 112 potentially causal variants in 107 probands (including four sib pairs) were reported to referring clinicians around the UK via DECIPHER (Supplementary Table 1). Nineteen reported variants were recurrent in two or more unrelated probands in our data set, including NM_001163213.1(FGFR3):c.749C>G (p.Pro250Arg) causing Muenke syndrome present in six unrelated DDD probands^16^ and NM_001197104.1(KMT2A):c.2318dup (p.Ser774ValfsTer12) causing Wiedemann–Steiner syndrome present in five unrelated DDD probands.

We judged the remaining 106 variants to be likely benign or incompletely penetrant in the heterozygous state, as they were either too common in gnomAD^4^ to be a plausible cause of a rare developmental disorder and/or the published phenotypes were not consistent with the child’s phenotypes or the child already has a diagnosis that fully explains their phenotypes (Supplementary Table 2). Of these, 21 were recurrent and 73/106 (69%) were inherited from an apparently unaffected parent. The MAF in gnomAD^4^ was an excellent predictor of whether we classed the variant as being potentially diagnostic, and we reported 90/136 (66%) variants with MAF < 0.000005 versus 21/82 (26%) with MAF >0.000005 (*p* < 0.0001). For example, we reviewed five variants in *LZTR1* present in 14 probands and reported none—three (recurrent) were present in 8, 17, and 21 individuals in gnomAD respectively, while two (one missense, one noncoding) did not fit the clinical phenotype in our probands and only had a single submitter (1* rating) in ClinVar. The gold star rating in ClinVar was also a good predictor of whether we classed a variant as being potentially diagnostic, and we reported 59/139 (42%) 1* variants versus 53/79 (67%) 2* and 3* variants (*p* = 0.0007) in our 218 additional variants. Interestingly, 53% of the 1134 ClinVar P/LP variants previously reported by the DDD study were 2* or 3* versus 47% of the 112 newly reported variants, suggesting the automated variant filtering pipeline is fairly sensitive for pathogenic variants.

## DISCUSSION

We were able to find 107 additional potential diagnoses in the DDD study by reanalyzing exome sequence data from 13,462 probands for known variants classified as P/LP in ClinVar that were missed by standard variant filtering. In 61/107 cases, the ClinVar variants are causative, while 27/107 partially explained the proband’s developmental disorder and 19/107 were considered uncertain but possibly diagnostic pending further clinical evaluation. Based on evaluation of the variants and patient phenotypes, we concluded that 106 P/LP variants with ≥1-star status in ClinVar appear not to be causing relevant phenotypes in our patients and are therefore likely to be either benign or incompletely penetrant in the heterozygous state. These findings reaffirm the importance of careful clinical review to ensure that the patient’s presentation fits with the genetic findings before establishing a definitive diagnosis.

As has been well documented previously, erroneous classifications of pathogenicity exist in most variant databases^17^ due to insufficient evidence being available at the time of classification. To reflect this variability in evidence levels, ClinVar provides a representation of the aggregate review status for a variant using gold stars (see https://www.ncbi.nlm.nih.gov/clinvar/docs/review_status/); thus, 1* variants have lower evidence associated with them than 2* or 3* variants. Nonetheless, many variant classifications predate the availability of population databases such as gnomAD,^4^ and some variants that were previously considered to be pathogenic should now be re-evaluated in light of their frequency in the population.^18^ This point is particularly relevant in the case of severe dominant developmental disorders, for which causal variants are heavily depleted from population databases.

Some of our findings have resulted in minor changes to the automated variant filtering pipeline using in the DDD study to ensure that similar variants are not missed in future. For example, including inherited variants in imprinted genes and paternally inherited X-chromosome variants in girls in the rare class of X-linked overdominant genes.^19^ In addition, the status of several genes in DDG2P has been recurated, either changing the mode of inheritance or removing the gene entirely as a cause of developmental disorders. However, the majority of automated rules that excluded these variants remain unchanged as they are important for maintaining the specificity of the pipeline and minimizing false positive results. For example, the majority of variants inherited from an unaffected parent are likely to be benign, and excluding these variants is one of the main reasons family trio data is so widely used for making diagnoses from exome sequencing data.^9^ Even by relaxing this variant exclusion rule only for known pathogenic variants, we still judged only 25% to be potentially diagnostic in our cohort. Moreover, of the 27 variants we reported that were inherited from an apparently unaffected parent, eight showed evidence of possible parental mosaicism (variant allele fraction <0.4 and child allele fraction >0.4).

Incompletely penetrant variants are of particular interest from a technical perspective because they would be included by standard variant filtering in singleton probands (for whom no parental genomic data is available), and thus represent a potential pitfall of the family trio approach. They may also represent a failure to accurately phenotype parents, some of whom may have or have had relevant phenotypes, albeit in a milder presentation or with different expressivity than the proband. Interestingly, we reported a higher proportion of inherited variants from apparently unaffected fathers than mothers (37% versus 19%, *p* = 0.07). Across the whole DDD cohort, 6% of fathers and 9% of mothers (*p* = 0.0001) have been annotated as having similar clinical features to the child (e.g., relevant HPO terms recorded in DECIPHER), suggesting a systematic underascertainment particularly of relevant paternal phenotypes. This observation fits with clinical experience that mothers are more likely to bring a child to clinic and are therefore more likely to have similar clinical features observed and recorded.

In summary, 1246 variants classified as pathogenic or likely pathogenic and with a ≥1-star status and no conflicts in ClinVar have now been communicated as possible diagnoses in 1204 (9%) probands in the DDD study. Of these, 107 (9%) diagnoses were missed by our standard variant filtering pipeline. We expect that further diagnoses could be found with a more complete list of known recurrent activating or dominant negative variants. Due to the scale of the DDD study, we deliberately adopted a fairly stringent automated variant filtering scheme, so it is likely that other pipelines with less stringent variant filtering would see less of an uplift in diagnoses. Nonetheless, some variant filtering choices are shared by most clinical genomic analysis pipelines; for example, noncoding variants and variants inherited from unaffected parents are frequently excluded and accounted for 38 (36%) of our additional diagnosis. We therefore recommend that other genome-wide sequencing studies and diagnostic services with similarly stringent automated workflows either perform a similar reanalysis or add a variant “inclusion” list of known pathogenic variants to their automated workflow for review.

## Supplementary information

Supplementary Table 1 and 2
